# Supplementary material for: “Gene accordions” cause genotypic and phenotypic heterogeneity in clonal populations of Staphylococcus aureus
Source: Nat Commun. 2020 Jul 14;11:3526. doi: 10.1038/s41467-020-17277-3 (PMC7360770; doi:10.1038/s41467-020-17277-3)
Supplement: Supplementary file 1 — Supplementary Information [file 41467_2020_17277_MOESM1_ESM.pdf]

Supplementary Information

Belikova et al. 2020

Gene accordions cause genotypic and phenotypic heterogeneity in clonal populations of *Staphylococcus aureus*

Supplementary Figure 1

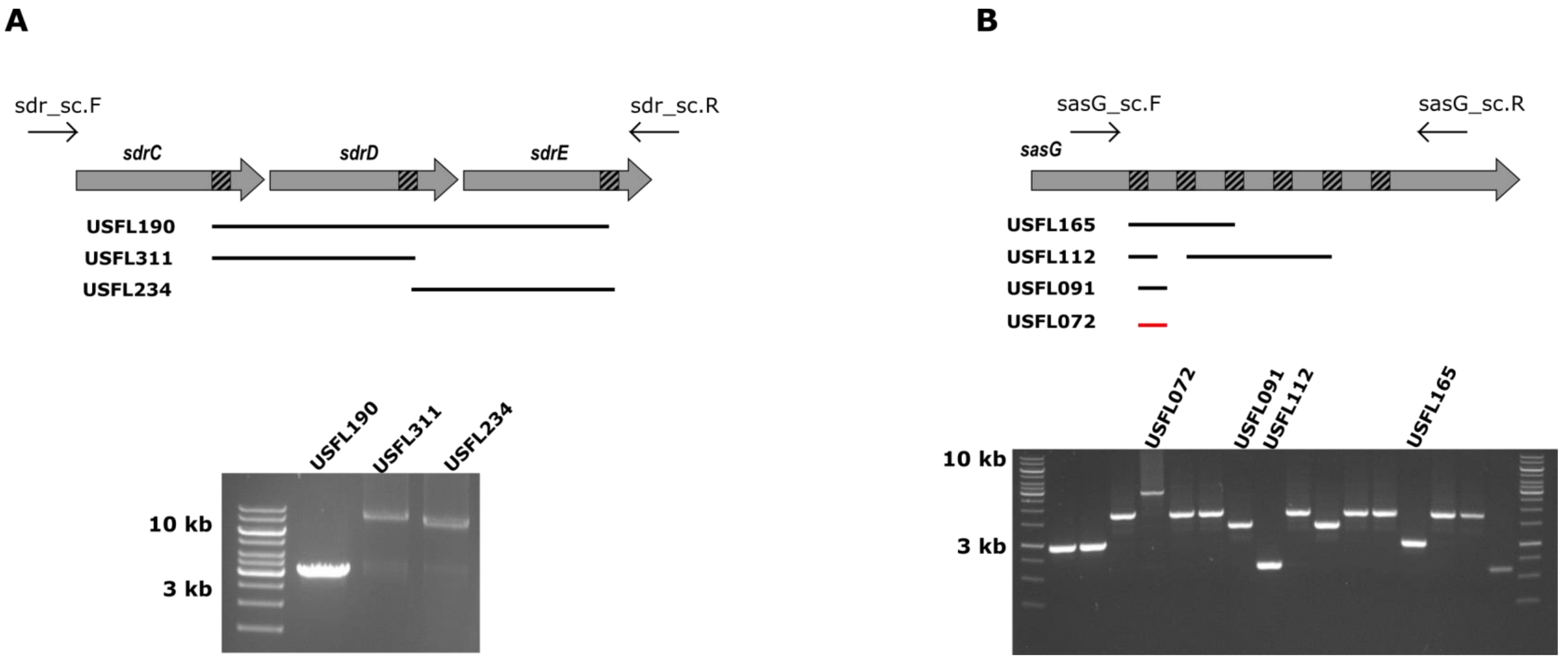

Supplementary Figure 1. Copy number variations in the *sdrCDE* locus and the *sasG* gene.
A) Schematic representation of the deletions within the *sdrCDE* loci for the indicated isolates. Coding sequences are shown and hatched boxes indicate repetitive DNA sequences encoding SD repeats. Black lines represent deletions within the USFL isolates. The lower panel shows DNA fragments amplified by conventional PCR using primers sdr_Sc.F and sdr_Sc.R. The experiment was performed once. Source data are provided as Source Data file.
B) Schematic representation of deletions and amplifications within *sasG*. Hatched boxes indicate G5-E domain encoding motifs. Black and red lines represent deletions and amplifications within the USFL-isolates, respectively. The lower panel shows DNA fragments amplified by conventional PCR using primers sasG_Sc.F and sasG_Sc.R. The experiment was performed once. Source data are provided as Source Data file.

Supplementary Figure 2

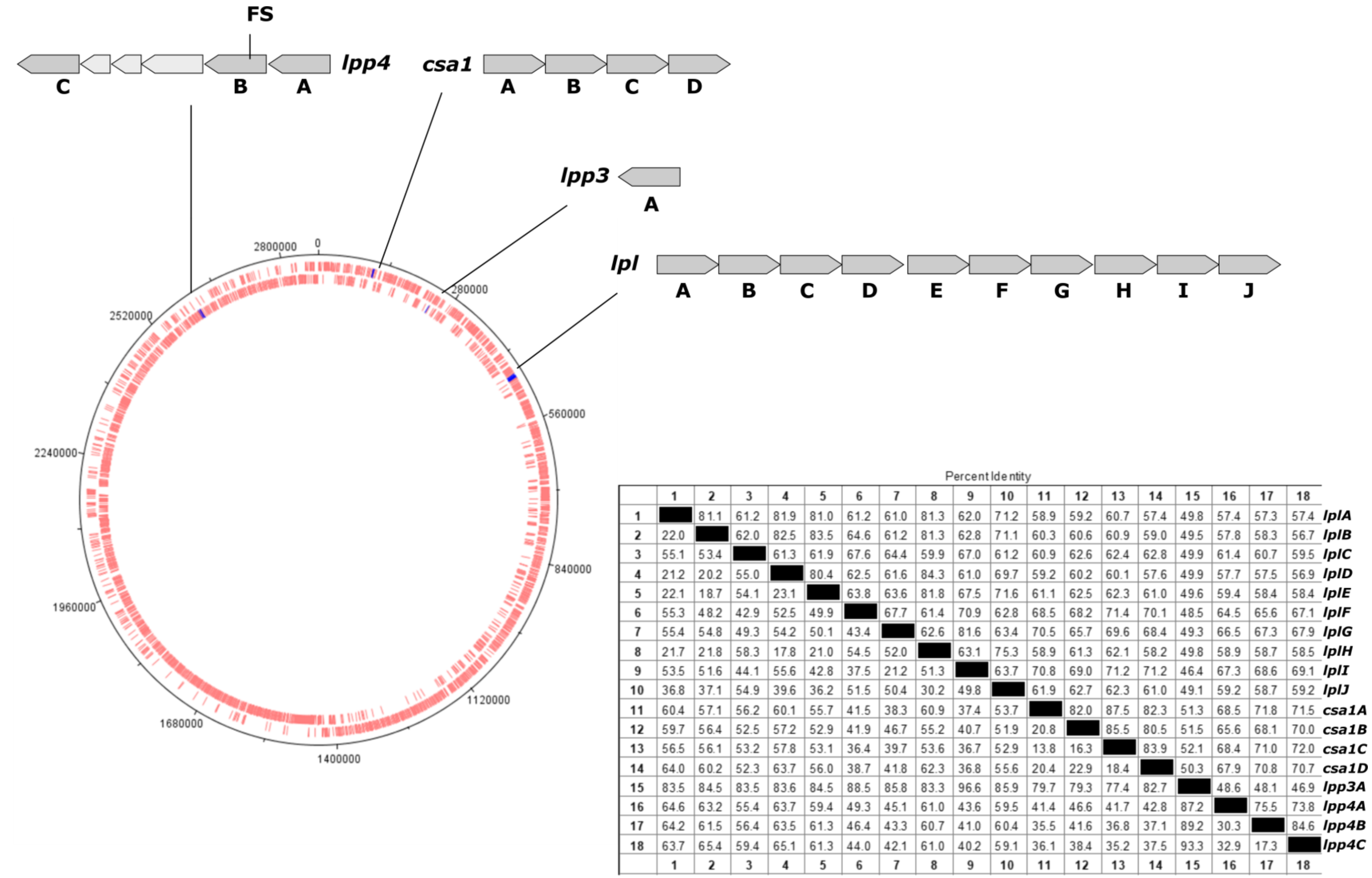

Percent Identity

	1	2	3	4	5	6	7	8	9	10	11	12	13	14	15	16	17	18	
1		81.1	61.2	81.9	81.0	61.2	61.0	81.3	62.0	71.2	58.9	59.2	60.7	57.4	49.8	57.4	57.3	57.4	***lplA***
2	22.0		62.0	82.5	83.5	64.6	61.2	81.3	62.8	71.1	60.3	60.6	60.9	59.0	49.5	57.8	58.3	56.7	***lplB***
3	55.1	53.4		61.3	61.9	67.6	64.4	59.9	67.0	61.2	60.9	62.6	62.4	62.8	49.9	61.4	60.7	59.5	***lplC***
4	21.2	20.2	55.0		80.4	62.5	61.6	84.3	61.0	69.7	59.2	60.2	60.1	57.6	49.9	57.7	57.5	56.9	***lplD***
5	22.1	18.7	54.1	23.1		63.8	63.6	81.8	67.5	71.6	61.1	62.5	62.3	61.0	49.6	59.4	58.4	58.4	***lplE***
6	55.3	48.2	42.9	52.5	49.9		67.7	61.4	70.9	62.8	68.5	68.2	71.4	70.1	48.5	64.5	65.6	67.1	***lplF***
7	55.4	54.8	49.3	54.2	50.1	43.4		62.6	81.6	63.4	70.5	65.7	69.6	68.4	49.3	66.5	67.3	67.9	***lplG***
8	21.7	21.8	58.3	17.8	21.0	54.5	52.0		63.1	75.3	58.9	61.3	62.1	58.2	49.8	58.9	58.7	58.5	***lplH***
9	53.5	51.6	44.1	55.6	42.8	37.5	21.2	51.3		63.7	70.8	69.0	71.2	71.2	46.4	67.3	68.6	69.1	***lplI***
10	36.8	37.1	54.9	39.6	36.2	51.5	50.4	30.2	49.8		61.9	62.7	62.3	61.0	49.1	59.2	58.7	59.2	***lplJ***
11	60.4	57.1	56.2	60.1	55.7	41.5	38.3	60.9	37.4	53.7		82.0	87.5	82.3	51.3	68.5	71.8	71.5	***csa1A***
12	59.7	56.4	52.5	57.2	52.9	41.9	46.7	55.2	40.7	51.9	20.8		85.5	80.5	51.5	65.6	68.1	70.0	***csa1B***
13	56.5	56.1	53.2	57.8	53.1	36.4	39.7	53.6	36.7	52.9	13.8	16.3		83.9	52.1	68.4	71.0	72.0	***csa1C***
14	64.0	60.2	52.3	63.7	56.0	38.7	41.8	62.3	36.8	55.6	20.4	22.9	18.4		50.3	67.9	70.8	70.7	***csa1D***
15	83.5	84.5	83.5	83.6	84.5	88.5	85.8	83.3	96.6	85.9	79.7	79.3	77.4	82.7		48.6	48.1	46.9	***lpp3A***
16	64.6	63.2	55.4	63.7	59.4	49.3	45.1	61.0	43.6	59.5	41.4	46.6	41.7	42.8	87.2		75.5	73.8	***lpp4A***
17	64.2	61.5	56.4	63.5	61.3	46.4	43.3	60.7	41.0	60.4	35.5	41.6	36.8	37.1	89.2	30.3		84.6	***lpp4B***
18	63.7	65.4	59.4	65.1	61.3	44.0	42.1	61.0	40.2	59.1	36.1	38.4	35.2	37.5	93.3	32.9	17.3		***lpp4C***
	1	2	3	4	5	6	7	8	9	10	11	12	13	14	15	16	17	18	

Supplementary Figure 2. Distribution of the *lpp* genes in the chromosome of *S. aureus* USA300 LAC. Shown is a circular diagram of the USA300 chromosome. The two concentric circles indicate both coding strands. Coding sequences are shown in pale red. Genes encoding Lpps are shown in blue and the corresponding loci magnified. The table on the right shows the percentage of identity between all the genes. FS: frame shift.

Supplementary Figure 3

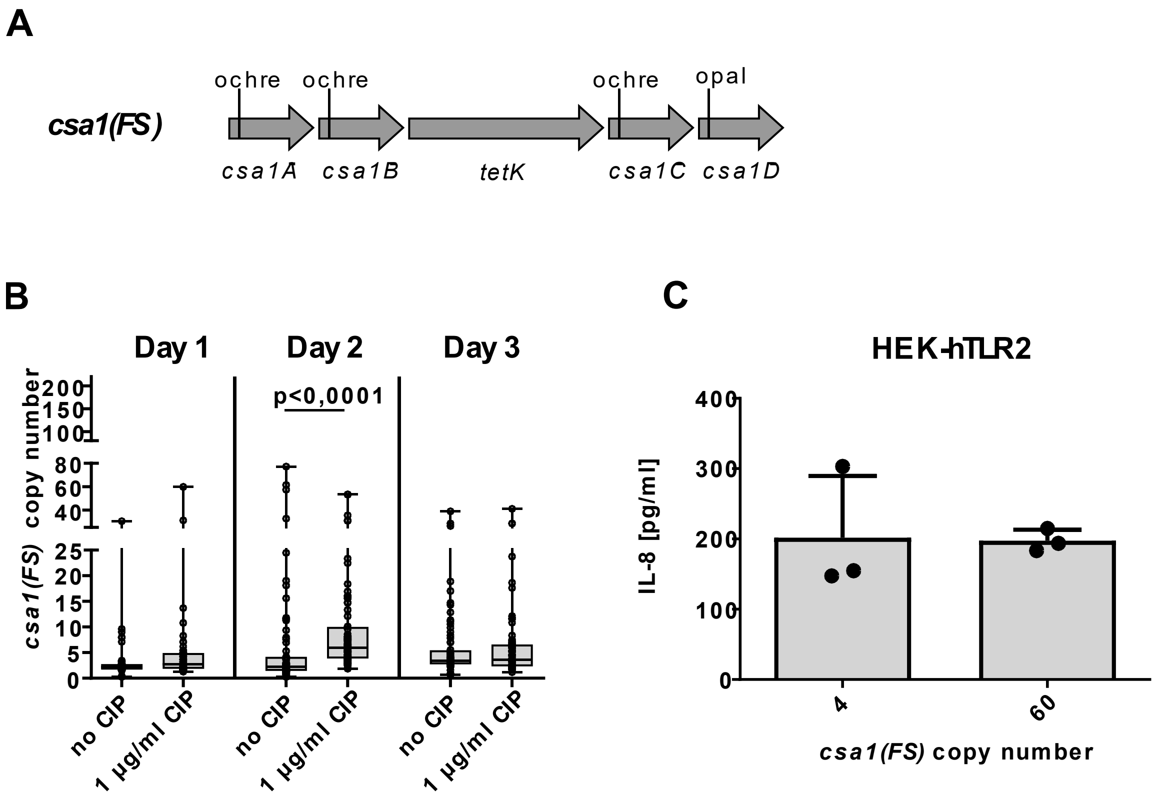

Supplementary Figure 3. Amplification of the inactivated *csa1⁻::tetK* locus.

A) Schematic representation of the *csa1(FS)::tetK*-locus. The position of the nonsense mutations in each *csa1* gene is indicated.

B) USA300 *csa1(FS)::tetK* was grown over three consecutive days in the presence or absence of 1 µg/ml ciprofloxacin (CIP). Each day, the copy number of up to 16 clones of each culture showing high Tc-resistance was screened by qPCR. Upper and lower box limits and the horizontal lines within the boxes represent 25 and 75% percentiles and the medians, respectively. The whiskers of the plots indicate minimum and maximum range. Data are derived from four independent experiments and represent: Day 1, n=60 and n=58 of "no Cip" and "1 µg/ml Cip", respectively; Day 2, n=70 and n=72 of "no Cip" and "1 µg/ml Cip", respectively; Day 3, n=72 and n=72 of "no Cip" and "1 µg/ml Cip", respectively. All data points are shown. Datasets were not Normal distributed (D'Agostino & Pearson omnibus test <0,0001) and statistical analysis was performed using the two-tailed Mann Whitney test. Source data are provided as Source Data file.

C) Confluent lawns of HEK-hTLR2 cells were stimulated for 18 h with 0.5 % culture filtrates of *csa1(FS)* copy number variants grown to stationary phase. IL-8 protein levels within the supernatants were quantified by ELISA (R&D Systems). Shown are the Mean and SD of three independent experiments. Statistical analysis was performed using students unpaired t-test (F=30,67; Dfn=2; Dfd=2), no differences were found. Source data are provided as Source Data file.
