# Supplementary material for: “Gene accordions” cause genotypic and phenotypic heterogeneity in clonal populations of Staphylococcus aureus
Source: Nat Commun. 2020 Jul 14;11:3526. doi: 10.1038/s41467-020-17277-3 (PMC7360770; doi:10.1038/s41467-020-17277-3)
Supplement: Supplementary file 3 — Description of Additional Supplementary Files [file 41467_2020_17277_MOESM3_ESM.pdf]

Description of Additional Supplementary Files

File Name: Supplementary Data 1
Description: Comparative genomic analysis of S. aureus USA300 clinical isolates
