# Supplementary material for: “Gene accordions” cause genotypic and phenotypic heterogeneity in clonal populations of Staphylococcus aureus
Source: Nat Commun. 2020 Jul 14;11:3526. doi: 10.1038/s41467-020-17277-3 (PMC7360770; doi:10.1038/s41467-020-17277-3)
Supplement: Supplementary file 5 — Reporting Summary [file 41467_2020_17277_MOESM5_ESM.pdf]

nature research

Corresponding author(s): Simon Heilbronner

Last updated by author(s): 05.06.2020

Reporting Summary

Nature Research wishes to improve the reproducibility of the work that we publish. This form provides structure for consistency and transparency in reporting. For further information on Nature Research policies, see Authors & Referees and the Editorial Policy Checklist .

Statistics

For all statistical analyses, confirm that the following items are present in the figure legend, table legend, main text, or Methods section.

n/a	Confirmed	
☐	☒	The exact sample size (*n*) for each experimental group/condition, given as a discrete number and unit of measurement
☐	☒	A statement on whether measurements were taken from distinct samples or whether the same sample was measured repeatedly
☐	☒	The statistical test(s) used AND whether they are one- or two-sided<br>*Only common tests should be described solely by name; describe more complex techniques in the Methods section.*
☒	☐	A description of all covariates tested
☐	☒	A description of any assumptions or corrections, such as tests of normality and adjustment for multiple comparisons
☐	☒	A full description of the statistical parameters including central tendency (e.g. means) or other basic estimates (e.g. regression coefficient) AND variation (e.g. standard deviation) or associated estimates of uncertainty (e.g. confidence intervals)
☐	☒	For null hypothesis testing, the test statistic (e.g. *F*, *t*, *r*) with confidence intervals, effect sizes, degrees of freedom and *P* value noted<br>*Give P values as exact values whenever suitable.*
☒	☐	For Bayesian analysis, information on the choice of priors and Markov chain Monte Carlo settings
☒	☐	For hierarchical and complex designs, identification of the appropriate level for tests and full reporting of outcomes
☒	☐	Estimates of effect sizes (e.g. Cohen's *d*, Pearson's *r*), indicating how they were calculated

Our web collection on statistics for biologists contains articles on many of the points above.

Software and code

Policy information about availability of computer code

Data collection	- Scaffolding analysis of short reads - SMALT (www.sanger.ac.uk/science/tools/smalt-0)<br>- qPCR data collection - QuantStudioTM Design and Analysis Software v1.4.1<br>- Infra Red Protein Quantification (LI-COR) - Image Studio Ver. 5.0<br>- Long Read Sequencing analysis - NUCmer (MUMmer v3.23, run using Bioconda v4.8.0
Data analysis	- Genome scaffolding analysis - SMALT (www.sanger.ac.uk/science/tools/smalt-0); Artemis Release 18.1.0<br>- Long Read Sequencing analysis - NUCmer (MUMmer v3.23 - https://anaconda.org/bioconda/mummer) run using Bioconda v4.8.0 8https://bioconda.github.io/)<br><br>- Diagrams and statistics - GraphPad Prism 8

For manuscripts utilizing custom algorithms or software that are central to the research but not yet described in published literature, software must be made available to editors/reviewers. We strongly encourage code deposition in a community repository (e.g. GitHub). See the Nature Research guidelines for submitting code & software for further information.

Data

Policy information about availability of data

All manuscripts must include a data availability statement. This statement should provide the following information, where applicable:

- Accession codes, unique identifiers, or web links for publicly available datasets
- A list of figures that have associated raw data
- A description of any restrictions on data availability

The datasets used and/or analysed during the current study are available as Source Data with this manuscript. MinION sequencing data are deposited in the NCBI

BioProject data-base under the accession number PRJNA601323w. USA300 datasets analysed were previously deposited in the European Nucleotide archive under accession number PRJEB2870.

Field-specific reporting

Please select the one below that is the best fit for your research. If you are not sure, read the appropriate sections before making your selection.

- [x] Life sciences
- [ ] Behavioural & social sciences
- [ ] Ecological, evolutionary & environmental sciences

For a reference copy of the document with all sections, see nature.com/documents/nr-reporting-summary-flat.pdf

Life sciences study design

All studies must disclose on these points even when the disclosure is negative.

Sample size	Sample sizes used in evolutionary experiments (Fig 2 and Fig 4) were based on the feasibility of the experimental workload. 6 parallel cultures ensured sufficient biological replication and 16 Clones screened at each day allowed sufficient to describe variation<br>Number of mice used for in vivo experiments were used in line with our allowance of animal numbers per group. Binding us to 6 mice per group. This is in our eyes sufficient to determine variation between individual mice.
Data exclusions	No datasets were excluded.
Replication	According to general practice, 3-4 technical replicates were performed e.g. for pPCR measurements. And 3-4 Biological replicates to discriminate day to day variation (e.g. for Stimulation of Cell lines, detection of Protein in the membranes...). Kind and number of replicates as well as success of replication are given in the figure legends.
Randomization	Allocation of mice to groups infected with different strains was random. For in vitro experiments groups were formed accoding to the identity of strains or experimental condition.
Blinding	Blinding of the investigator during in vitro experimentation was not performed. Experimenters planned an conducted experiments individually and were therefore aware of identity of strains analysed.<br>Blinding for in vivo experiments was performed as the experimenter was not informed about the strain the individual mice were infected with.

Reporting for specific materials, systems and methods

We require information from authors about some types of materials, experimental systems and methods used in many studies. Here, indicate whether each material, system or method listed is relevant to your study. If you are not sure if a list item applies to your research, read the appropriate section before selecting a response.

Materials & experimental systems

n/a	Involved in the study	
[ ]	[x]	Antibodies
[ ]	[x]	Eukaryotic cell lines
[x]	[ ]	Palaeontology
[ ]	[x]	Animals and other organisms
[ ]	[x]	Human research participants
[x]	[ ]	Clinical data

Methods

n/a	Involved in the study	
[x]	[ ]	ChIP-seq
[x]	[ ]	Flow cytometry
[x]	[ ]	MRI-based neuroimaging

Antibodies

Antibodies used	mouse anti-Csa1C serum (1:1000), rabbit anti-RecA (1:3000) (Abcam, ab63797), goat α-mouse-DYLight 800 antibodies (LI-COR 926-32211) , rabbit anti-SdrD serum (1:1000) (kind gift of T.J. Foster) , goat α-rabbit-DYLight 800 secondary antibodies (LICOR 926-32211)
Validation	anti-Csa1C serum, which was used for Western blot analysis, was described in "Schluepen C, Malito E, Marongiu A, Schirle M, McWhinnie E, Lo Surdo P, Biancucci M, Falugi F, Nardi-Dei V, Marchi S et al: Mining the bacterial unknown proteome: identification and characterization of a novel family of highly conserved protective antigens in Staphylococcus aureus. Biochem J 2013, 455(3):273-284".<br><br>Anti-SdrD serum was used for Western blot analysis.<br><br>Specificity of all the primary antibodies was confirmed on strains lacking the proteins that are recognized by the antibodies.

Eukaryotic cell lines

Policy information about cell lines

Cell line source(s)	HEK cells stably transfected with the human TLR2 gene were purchased from Invivogen.<br><br>HL60 cells were purchased from DSMZ.
Authentication	No authentication was performed
Mycoplasma contamination	All cell lines were tested negative for mycoplasma contamination
Commonly misidentified lines<br>(See ICLAC register)	No commonly misidentified cell lines were used in the study.

Animals and other organisms

Policy information about studies involving animals; ARRIVE guidelines recommended for reporting animal research

Laboratory animals	Six-week old female C57BL/6 wild-type mice purchased from Envigo. Housing conditions are described in the methods section.
Wild animals	No wild animals were used in the study.
Field-collected samples	No field collected samples were used in the study.
Ethics oversight	Animal experiments were performed in strict accordance with the European Health Law of the Federation of Laboratory Animal Science Associations. The protocol was approved by the Regierungspräsidium Tübingen (IMIT1/17)

Note that full information on the approval of the study protocol must also be provided in the manuscript.

Human research participants

Policy information about studies involving human research participants

Population characteristics	All participants were older than 18 years. Gender, age, or genotypic information was not recorded and controlled
Recruitment	Students and associates of our University were randomly and spontaneously recruited. This might have led to a bias towards rather young participants (in their 20s) working as PhD students. However, we assume that this bias is unlikely to affect the results of our study.
Ethics oversight	Human PMNs were isolated from venous blood of healthy volunteers in accordance with protocols approved by the Institutional Review Board for Human Subjects at the University of Tübingen. Informed written consent was obtained from all volunteers.

Note that full information on the approval of the study protocol must also be provided in the manuscript.
