# Supplementary figures and images for: “Gene accordions” cause genotypic and phenotypic heterogeneity in clonal populations of Staphylococcus aureus
Source: Nat Commun. 2020 Jul 14;11:3526. doi: 10.1038/s41467-020-17277-3 (PMC7360770; doi:10.1038/s41467-020-17277-3)

**Figure 1C: full blot of PCR of *csa1* in USFL strains**

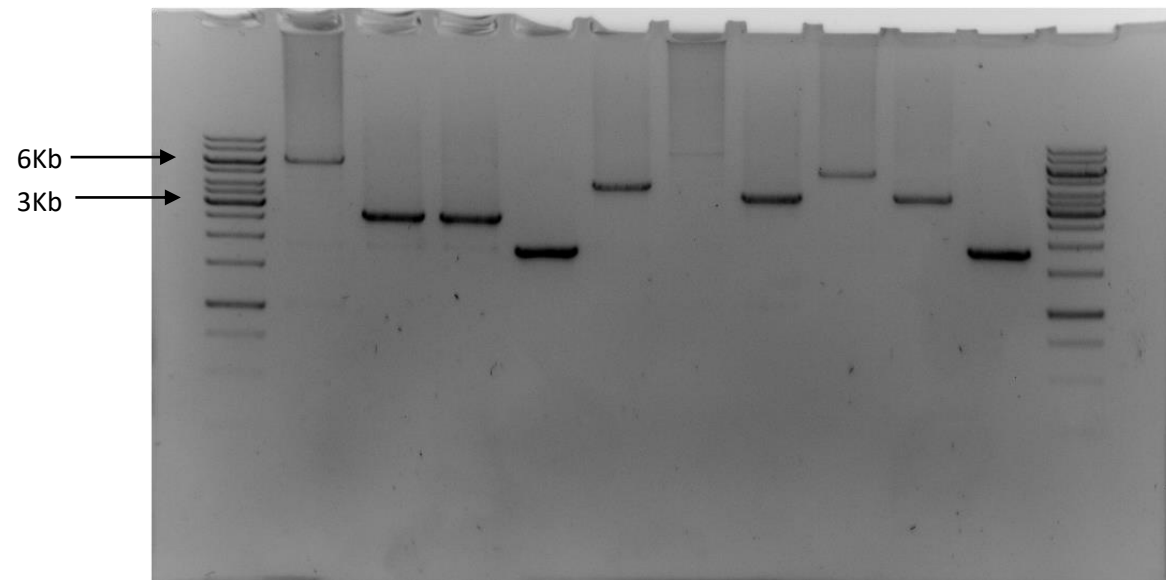

Supplement: Supplementary file 6 — Source Data [file 41467_2020_17277_MOESM6_ESM.zip › Submission folder/Source Data Fig.1C_agarose gel.pdf]

**Figure 5A. Csa1 protein expression**

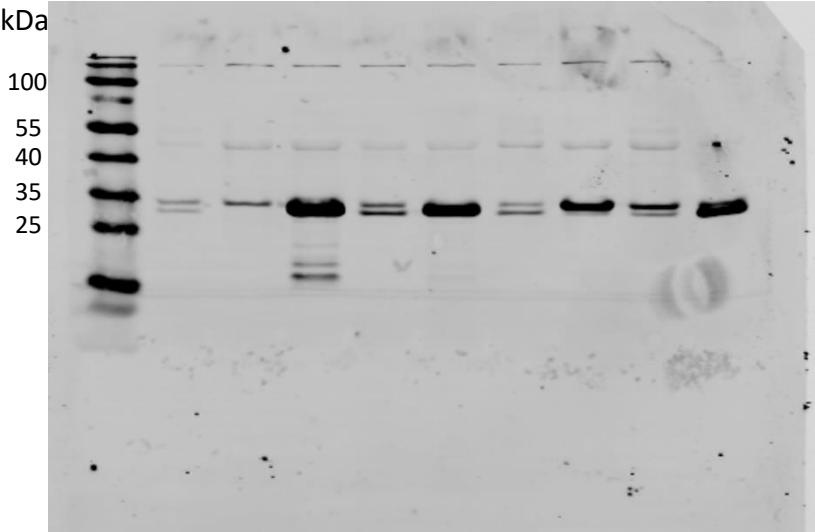

**Figure 5B. SdrD protein expression**

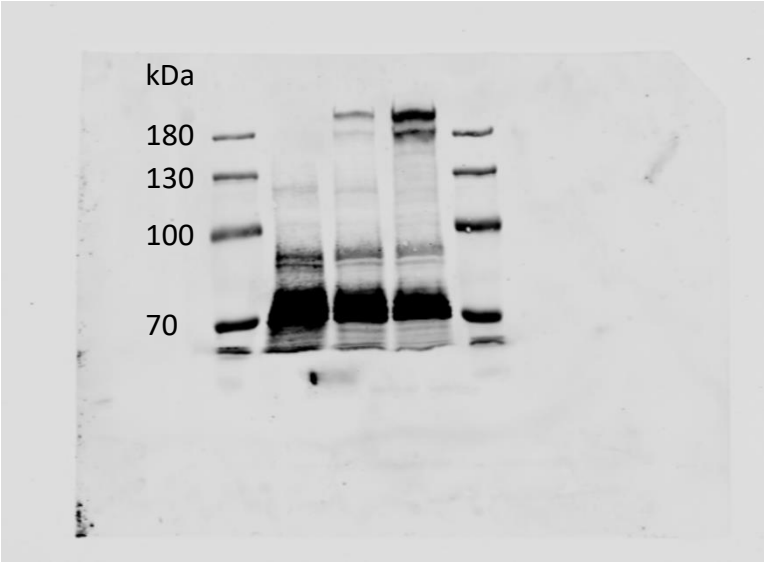

Supplement: Supplementary file 6 — Source Data [file 41467_2020_17277_MOESM6_ESM.zip › Submission folder/Source Data Figure 5_blots.pdf]

**Figure 2B. RecA protein induction**

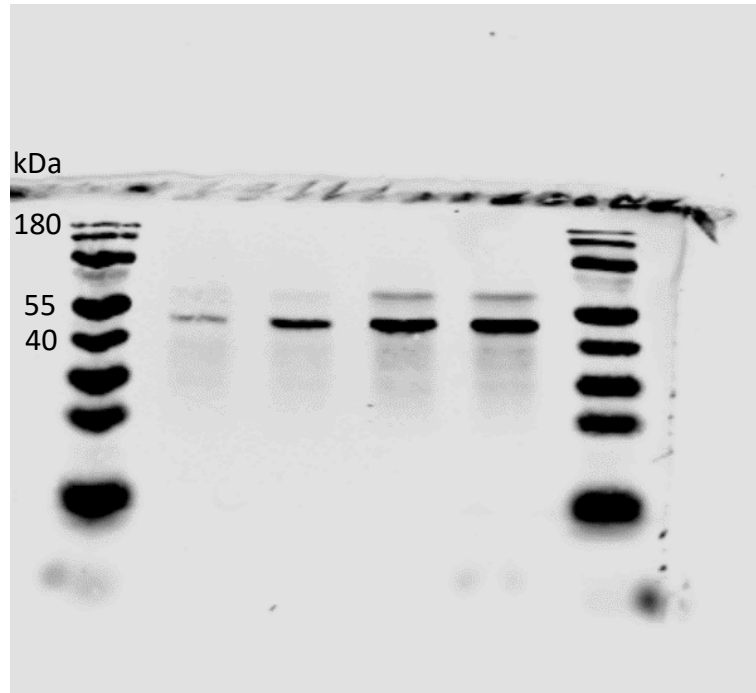

Supplement: Supplementary file 6 — Source Data [file 41467_2020_17277_MOESM6_ESM.zip › Submission folder/Source Data Figure2B_RecAblot.pdf]

**Supplementary Figure 1A: SdrD of USFL strains**

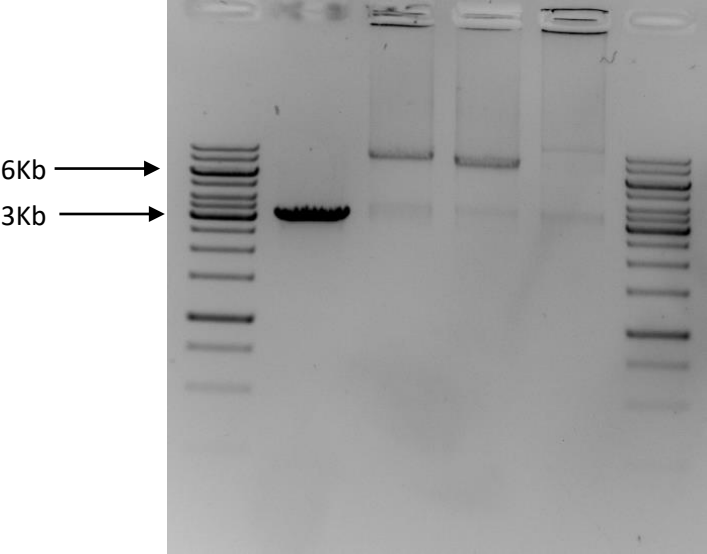

**Supplementary Figure 1B: SasG of USFL strains**

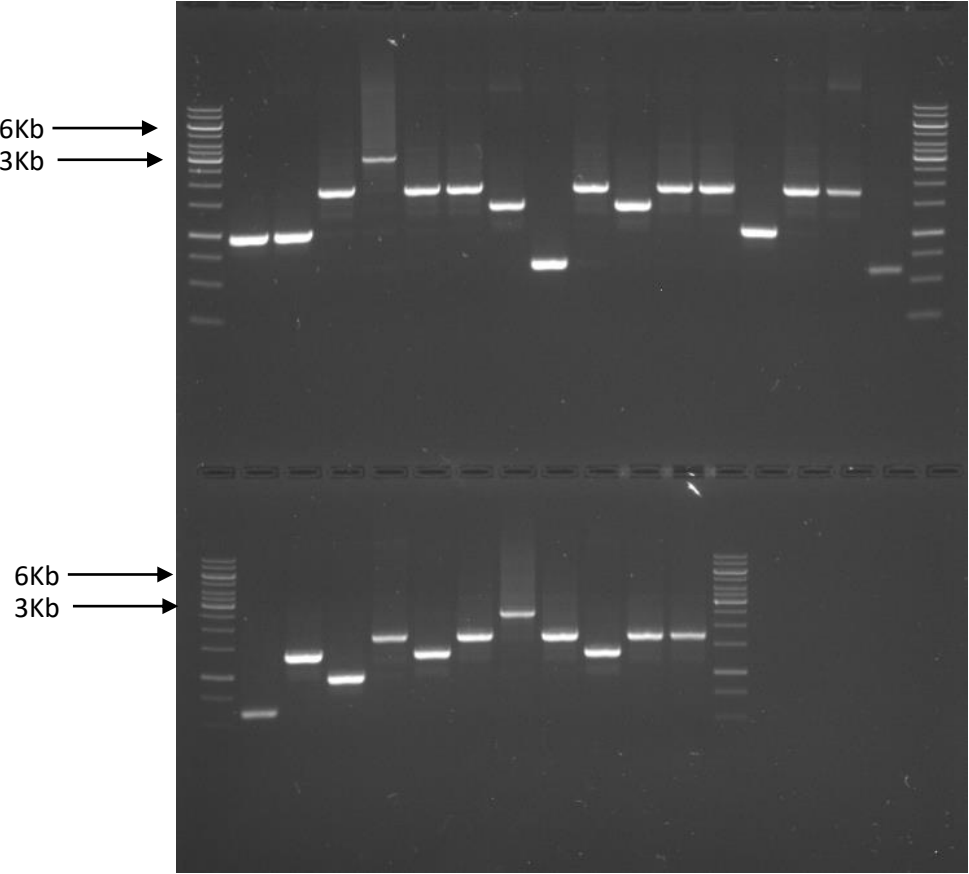

Supplement: Supplementary file 6 — Source Data [file 41467_2020_17277_MOESM6_ESM.zip › Submission folder/Source Data Supplementary Figure 1_Agarose gels.pdf]
